# The Role of Arginase and Rho Kinase in Cardioprotection from Remote Ischemic Perconditioning in Non-Diabetic and Diabetic Rat *In Vivo*

**DOI:** 10.1371/journal.pone.0104731

**Published:** 2014-08-20

**Authors:** Attila Kiss, Yahor Tratsiakovich, Adrian T. Gonon, Olga Fedotovskaya, Johanna T. Lanner, Daniel C. Andersson, Jiangning Yang, John Pernow

**Affiliations:** 1 Division of Cardiology, Department of Medicine, Karolinska Institutet, Karolinska University Hospital, Stockholm, Sweden; 2 Division of Clinical Physiology, Department of Laboratory Medicine, Karolinska Institutet, Karolinska University Hospital, Huddinge, Sweden; 3 Department of Physiology and Pharmacology, Karolinska Institutet, Stockholm, Sweden; University of Missouri, United States of America

## Abstract

**Background:**

Pharmacological inhibition of arginase and remote ischemic perconditioning (RIPerc) are known to protect the heart against ischemia/reperfusion (IR) injury.

**Purpose:**

The objective of this study was to investigate whether (1) peroxynitrite-mediated RhoA/Rho associated kinase (ROCK) signaling pathway contributes to arginase upregulation following myocardial IR; (2) the inhibition of this pathway is involved as a cardioprotective mechanism of remote ischemic perconditioning and (3) the influence of diabetes on these mechanisms.

**Methods:**

Anesthetized rats were subjected to 30 min left coronary artery ligation followed by 2 h reperfusion and included in two protocols. In protocol 1 rats were randomized to 1) control IR, 2) RIPerc induced by bilateral femoral artery occlusion for 15 min during myocardial ischemia, 3) RIPerc and administration of the nitric oxide synthase inhibitor N^G^-monomethyl-L-arginine (L-NMMA), 4) administration of the ROCK inhibitor hydroxyfasudil or 5) the peroxynitrite decomposition catalyst FeTPPS. In protocol 2 non-diabetic and type 1 diabetic rats were randomosed to IR or RIPerc as described above.

**Results:**

Infarct size was significantly reduced in rats treated with FeTPPS, hydroxyfasudil and RIPerc compared to controls (P<0.001). FeTPPS attenuated both ROCK and arginase activity (P<0.001 vs. control). Similarly, RIPerc reduced arginase and ROCK activity, peroxynitrite formation and enhanced phospho-eNOS expression (P<0.05 vs. control). The cardioprotective effect of RIPerc was abolished by L-NMMA. The protective effect of RIPerc and its associated changes in arginase and ROCK activity were abolished in diabetes.

**Conclusion:**

Arginase is activated by peroxynitrite/ROCK signaling cascade in myocardial IR. RIPerc protects against IR injury via a mechanism involving inhibition of this pathway and enhanced eNOS activation. The beneficial effect and associated molecular changes of RIPerc is abolished in type 1 diabetes.

## Introduction

Emerging evidence suggested a pathophysiological role of arginase in several diseases related to endothelial dysfunction such as atherosclerosis [1], diabetes mellitus (DM) [2] and myocardial ischemia/reperfusion (IR) injury [3], [4], [5] by reducing bioavailability of nitric oxide (NO). Arginase regulates NO bioavailability by competing with endothelial NO synthase (eNOS) for their common substrate L-arginine. Arginase exists in two isoforms (1 and 2) and both are widely expressed in the cardiovascular system [6], [7]. Recently, our group has demonstrated that inhibition of arginase either prior to ischemia or reperfusion protects the heart against IR injury through NOS- and NO-dependent mechanisms [3], [4], [5]. These studies also confirmed that arginase activity is increased within the myocardium at risk after myocardial IR. Cell culture experiments have revealed that the expression and activity of arginase are regulated by several factors including pro-inflammatory cytokines, hypoxia, reactive oxygen and nitrogen species and RhoA/Rho associated kinase (ROCK) [7], [8], [9]. However, the mechanism underlying the upregulation of arginase activity in myocardial IR still remains to be identified.

It is well known that ischemic preconditioning provides a robust intrinsic cardioprotection in the experimental setting of myocardial IR [10]. The clinical application of preconditioning is limited, however, due to the unpredictable occurrence of myocardial infarction. More recently, it was described that remote ischemic perconditioning (RIPerc), defined as brief cycle(s) of IR of a remote organ applied during sustained myocardial ischemia also reduces myocardial infarct size [11], [12]. The magnitude of cardioprotection induced by RIPerc is comparable to preconditioning [12] but has the advantage of being feasible in the clinical setting on patients with ST elevation myocardial infarction [13]. It is still unclear how the protective signal translates from the remote organ to the heart and which signaling pathways within the myocardium that are involved as a mediator of the protection. Substantial evidence suggests that the attenuation of the deleterious effect of ROCK and the activation of eNOS are involved in the cardioprotective effect of RIPerc [14], [15]. Based on the link between ROCK and arginase activity described above, we hypothesized that the cardioprotective effect of RIPerc is associated with down-regulation of ROCK activity and arginase activity.

Recent studies have suggested that co-morbidities such as diabetes attenuate the cardioprotective effect of local pre- and postconditioning, at least in part, via attenuation of the eNOS signaling pathway in the myocardium [16], [17]. However, it is unknown, whether the molecular signaling and the cardioprotective effect to RIPerc is affected by co-morbidities.

Therefore, the present study was design to investigate (1) the contribution of oxidative/nitrosative stress and ROCK signaling pathway to IR-induced arginase upregulation and (2) whether inhibition of this pathway is involved in the cardioprotective mechanism of RIPerc in the absence and presence of diabetes.

## Materials and Methods

### 2.1 Ethics Statement

The study was approved by the regional Ethics Committee (Stockholm Norra Djurförsöksetiska Nämnd, approval number N192/12) for laboratory animal experiments in Stockholm and conforms to the Guide for the Care and Use of Laboratory Animals published by the US National Institutes of Health (NIH Publication No. 85-23, revised 1996).

### 2.2 Surgical procedures

The surgical interventions were similar as described previously [3], [5]. Male Sprague–Dawley rats (Charles-River, Sulzfeld, Germany) were anaesthetized with sodium pentobarbital (50 mg/kg ip, followed by 3–5 mg/kg/h iv throughout the experiment), tracheotomized, intubated and ventilated with air by a rodent ventilator (54 strokes/min, 9 ml/kg tidal volume). Rectal temperature was maintained at 37.5–38.5°C by a heated operating table. The right carotid artery was cannulated and connected to a pressure transducer for measurement of mean arterial pressure (MAP). Heart rate (HR) was determined from the arterial pressure curve. Hemodynamic parameters were continuously recorded on a personal computer equipped with PharmLab V5.0 (AstraZeneca R&D, Mölndal, Sweden). The left jugular vein was cannulated for drug administration. The heart was exposed via a left thoracotomy and a ligature was placed around the left coronary artery. Myocardial ischemia was induced by tightening of the ligature and successful occlusion was associated with cyanosis of the myocardial area at risk. Reperfusion was initiated after 30 min of ischemia by removal of the snare and was maintained for 2 h. The reperfusion was associated with disappearance of the cyanotic color of the myocardium. All efforts were made to minimize animals' suffering.

### 2.3 Induction of type 1 diabetes mellitus

Type 1 diabetes mellitus was induced by a single iv (tail) injection of streptozotocin (55 mg/kg, Sigma Aldrich, USA) [18]. Three days later, only rats with blood glucose levels >15.0 mM were considered to be diabetic. The rats were given unlimited food and water and were not supplemented with insulin or anti-hyperglycaemic agents. Four to five weeks following streptozotocin injection, the rats were used for IR experiments as described above. Age matched rats served as a non-diabetic control group.

### 2.4 Experimental protocols

#### 2.4.1 Protocol 1

Protocol 1 was designed to investigate whether the peroxynitrite/ROCK signaling pathway play role in arginase activation and whether the inhibition of this pathway is involved in the cardioprotective mechanism of RIPerc.

After the surgical preparation rats were allowed to stabilize for 15 min and randomized into one of the following groups: (1) control IR (CIR; no intervention during IR; n = 10), (2) RIPerc induced by bilateral femoral artery occlusion using vessels clamps during the last 15 min of coronary artery occlusion (n = 10), (3) administration of the NO synthase inhibitor N^G^-monomethyl-L-arginine inhibitor (L-NMMA, Alexis Biochemicals, Switzerland; 10 mg/kg, iv n = 6) just prior to RIPerc, (4) administration of the ROCK inhibitor hydroxyfasudil (Tocris Biosience, UK; 0.5 mg/kg, iv n = 7) 20 min prior to ischemia, or (5) administration of the peroxynitrite decomposition catalyst 5,10,15,20-tetrakis(4-sulfonatophenyl)porphyrinato iron (III), chloride (FeTPPS, Calbiochem, USA; 10 mg/kg, iv n = 6) were given 10 min prior to reperfusion. The dosage of drugs and the length of time the femoral artery occlusion were based on previous studies [5], [12], [19], [20]. NOS blockade has previously been demonstrated not to affect infarct size per se in our laboratory [21], [22], [23].

#### 2.4.2 Protocol 2

Protocol 2 aimed to assess the influence of diabetes on the cardioprotective effect and molecular signaling induced by RIPerc. After the surgical preparation as described above, type 1 diabetic or age-matched non-diabetic rats were allowed to stabilize for 15 min and randomized into four groups. These groups were: (1) non-diabetic controls (n = 6) with no intervention during IR (ND-CIR), (2) non-diabetic+RIPerc (ND-RIPerc; n = 6); (3) diabetic with no intervention during myocardial IR (DM-CIR; n = 7) and (4) diabetic+RIPerc (DM-RIPerc; n = 6).

### 2.5 Assessment of myocardial infarct size

Infarct size was measured as described previously [5]. Briefly, after 2 h of reperfusion, the coronary artery was reoccluded and 1.5 ml of 2% Evans Blue was injected in the right atrium via the left jugular vein to demarcated the area at risk (ischemic myocardium). Then rats were euthanized rapidly by exsanguination and the heart was rapidly excised. The atria and the right ventricle were removed. The left ventricle of hearts were frozen for 20 minutes (−20°C) and cut into 5–7 slices perpendicular to the base-apex axis. The third slice (counting from the apex) was cut into the ischemic and non-ischemic parts and frozen at −80°C for further analyses [5]. The remaining slices were scanned from both sides for determination of the area at risk weighed, and put in 1% triphenyltetrazolium chloride (TTC) for 15 min at 37°C to distinguish the viable myocardium from the necrotic. After 24 h of incubation in 4% formaldehyde, slices were again scanned from both sides. Viable myocardium is stained red by TTC, whereas necrotic myocardium appears pale yellow. The area at risk and the necrotic area were determined by computerized planimetry images (Photoshop 6.0; Adobe Systems, USA), normalized to the weight of each slice, with the degree of necrosis expressed as the percentage of area at risk.

### 2.6 Western blotting

Tissue samples from the left ventricle were extracted in lysate buffer (containing 20 mM KCl, 1 mM MgCl_2_, 0.5 mM Na_3_VO_4_, glycerol, 0.05 M NaF, 100 mM Na-pyrophosphate, Tris pH 7.8, Triton-X (0.1%), protease inhibitor (Roche), PBS and 1 mM of EDTA), homogenized and centrifuged for 20 min at 10000 g at 4°C. Total protein content of the extracts was quantified by using bicinchoninic acid protein assay kit (Pierce Biotechnology, USA). The proteins were separated on 7.5% SDS gel (10 or 40 µg/lane) and transferred onto either polyvinylidine fluoride (Millipore, USA) or nitrocellulose membranes (Amersham Biosciences, USA). Membranes were blocked for 1 h at room temperature and incubated overnight at 4°C with primary antibodies against arginase 1 and 2 (1∶1000, Sigma Prestige Antibody, USA); phoshorylated eNOS (p-eNOS; anti-phosphorylated Ser-1177; 1∶1000; BD Pharmigen, USA), total eNOS (1∶1000; ABR Affinity BioReagents, USA); 3-nitrotyrosine (3NT, 1∶1000; Abcam) or phosphorylated ezrin (p-ezrin; anti-phosphorylated Thr567, 1∶1000, BD Pharmingen, USA). IRDye 800-conjugated goat anti-mouse IgG (1∶12000, LI-COR), IRDye 800-conjugated goat anti-rabbit IgG (1∶12000, LI-COR) and IRDye 680 (1∶10000) used as secondary antibody and bands visualized using infrared fluorescence scanner (IR-Odyssey, LI-COR Biosciences, USA). Equal loading was confirmed by expression of GAPDH (1∶5000, Sigma Aldrich, USA) or, for 3NT, staining with Coomassie brilliant blue. Band densities were analyzed with Image Studio Lit Version 3.1 (LI-COR Biosciences, USA) and expressed percent of controls.

### 2.7 Determination of ROCK and arginase activity

Activity of ROCK was determined similar to described previously [14]. Briefly, left ventricular tissue samples were prepeared as described above. To evaluate Rho-kinase activity, the extent of phosphorylation of ezrin (Thr567) was determined by Western blotting.

Arginase activity was determined by using a modified colorimetric assay previously described [24]. The assay measures the urea content using α-isonitrosopropiophenone. Tissue samples from the area at risk and non-risk of the left ventricle were homogenized and centrifuged (see above), 50 µl of the supernatant was added to 75 µl of Tris-HCl (50 mM, pH 7.5) containing 10 mM MnCl_2_. The mixture was activated by heating for 10 min at 56°C. Each sample was then incubated at 37°C for 1 h with: (1) L-arginine (50 µl, 0.05 M, in Tris-HCl pH 9.7) and (2) L-arginine with 30 min preincubation with the arginase inhibitor 2 (S)-amino-6-boronohexanoic acid (100 µM; Enzo Clinical Labs, Farmingdale, NY, USA). The reaction was stopped by adding 400 µl of an acid solution (H_2_SO_4_–H_3_PO_4_–H_2_O = 1∶3∶7). 25 µl of α-isonitrosopropiophenone (9% in ethanol), and the mixture was then heated at 100°C for 60 min. Arginase activity was calculated as urea (µmol/mg protein/min) production and expressed in percent of control.

### 2.8 Statistical analysis

Data are presented as mean ± SEM. For multiple comparisons between several groups one-way ANOVA followed by Bonferroni post hoc test was used (Prism 5 software, GraphPad Inc., USA). Mann Whitney U-test was used for comparisons of only two groups where appropriate. The *P*-value<0.05 was considered to be significant.

## Results

### 3.1 Hemodynamics

Hemodynamic parameters of animals included in protocol 1 and 2 are presented in Table 1 and 2. There was no significant difference in MAP or HR between the groups in protocol 1 (Table 1). In protocol 2, baseline HR was significantly lower in rats with diabetes in comparison with the non-diabetic controls (Table 2).

**Table 1:** Hemodynamic changes in Protocol 1.

GROUP	Parameters	30 min before ischemia	Ischemia start	Ischemia 30 min	Reperfusion 30 min	Reperfusion 60 min	Reperfusion 120 min
**CIR**	**MABP**	90±5	92±5	79±3	80±6	86±6	71±5
	**HR**	420±8	420±11	412±10	410±13	403±11	389±10
**RIPerc**	**MABP**	88±4	93±5	76±4	75±4	76±3	72±2
	**HR**	419±7	421±10	406±10	399±8	390±9	375±7
**FeTPPS**	**MABP**	89±3	97±4	77±4	87±5	79±3	70±3
	**HR**	422±21	418±23	398±10	420±14	411±13	391±12
**H.fasudil**	**MABP**	94±6	90±4	77±2	89±8	84±8	77±3
	**HR**	409±10	406±7	397±13	377±4	377±3	366±5
**L-NMMA+RIPerc**	**MABP**	96±5	100±5	89±3	86±6	80±5	74±4
	**HR**	415±13	429±7	412±8	397±16	391±10	372±6

Values are mean ± SEM; n = 6–10. Abbreviations: MABP (mm Hg), mean arterial blood pressure; HR (beats/min), heart rate; CIR, control ischemia/reperfusion; RIPerc, remote ischemic perconditioning; FeTPPS, 5,10,15,20-Tetrakis(4-sulfonatophenyl)porphyrinato iron (III), chloride; H.fasudil, hydroxyfasudil and L-NMMA, N^G^-monomethyl-L-arginine.

**Table 2:** Hemodynamic changes in Protocol 2.

GROUP	Parameters	Baseline	Ischemia 30 min	Reperfusion 30 min	Reperfusion 60 min	Reperfusion 120 min
**ND- CIR**	**MABP**	92±2	77±6	85±6	82±6	77±2
	**HR**	422±11	391±8	384±9	385±7	379±6
**ND- RIPerc**	**MABP**	98±3	82±7	82±3	85±5	75±3
	**HR**	414±9	388±10	387±15	382±9	374±11
**DM- CIR**	**MAB**	97±8	82±2	82±2	77±6	72±5
	**HR**	363±6*	334±7*	336±6*	328±7*	320±2*
**DM- RIPerc**	**MABP**	97±4	80±4	83±3	79±5	77±2
	**HR**	360±4*	340±6*	347±13*	345±8*	328±10*

Values are mean ± SEM; n = 6–7. Abbreviations: MABP (mm Hg), mean arterial blood pressure; HR (beats/min), heart rate; ND-CIR, non-diabetic control ischemia/reperfusion; ND-RIPerc, non-diabeic remote ischemic perconditioning; DM-CIR: diabetes mellitus control ischemia/reperfusion; DM-RIPerc, diabetes mellitus remote perconditioning.

*P<0.05 vs. ND-CIR.

### 3.2 Results of protocol 1

#### 3.2.1 Effect of peroxynitrite and ROCK on arginase and ROCK activity in IR

**Figure 1:**
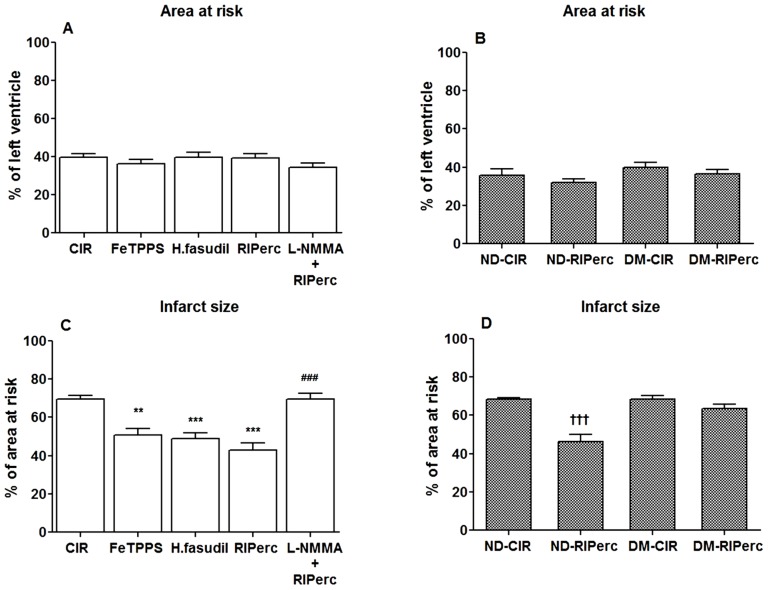
Myocardial area at risk and infarct size. Area at risk (A and B) expressed as % of left ventricle and infarct size (C and D) expressed as % of the area at risk following 30 min ischemia and 2 hrs reperfusion in rats included in protocol 1 (A and C) and in non-diabetic and diabetic rats included in protocol 2 (B and D). Values are means ± SEM; n = 6–10. **P<0.01, ***P<0.001 vs. CIR; ^###^ *P*<0.001 vs. RIPerc and ^†††^ *P*<0.001 vs. ND-CIR.

**Figure 2:**
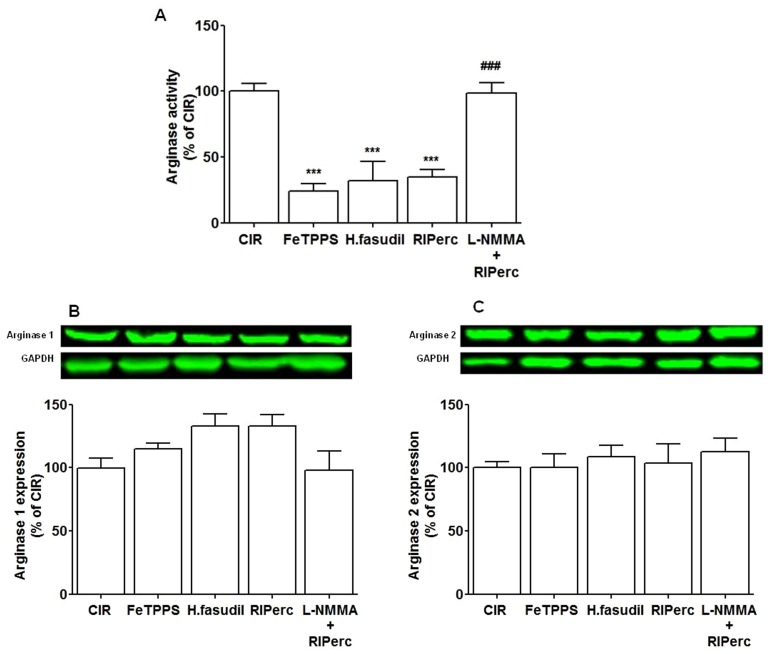
Arginase activity and protein expression in protocol 1. (A) Administration of the peroxynitrite decomposition catalyst FeTPPS, the ROCK inhibitor hydroxyfasudil (H.fasudil), remote ischemic preconditioning (RIPerc) and RIPerc+L-NMMA on arginase activity following ischemia/reperfusion. (B and C) Arginase 1 and 2 protein expression is depicted as representative blots. Values are means ± SEM n = 5–10. ***P<0.001 vs. CIR; ^###^ *P*<0.001 vs. RIPerc.

**Figure 3:**
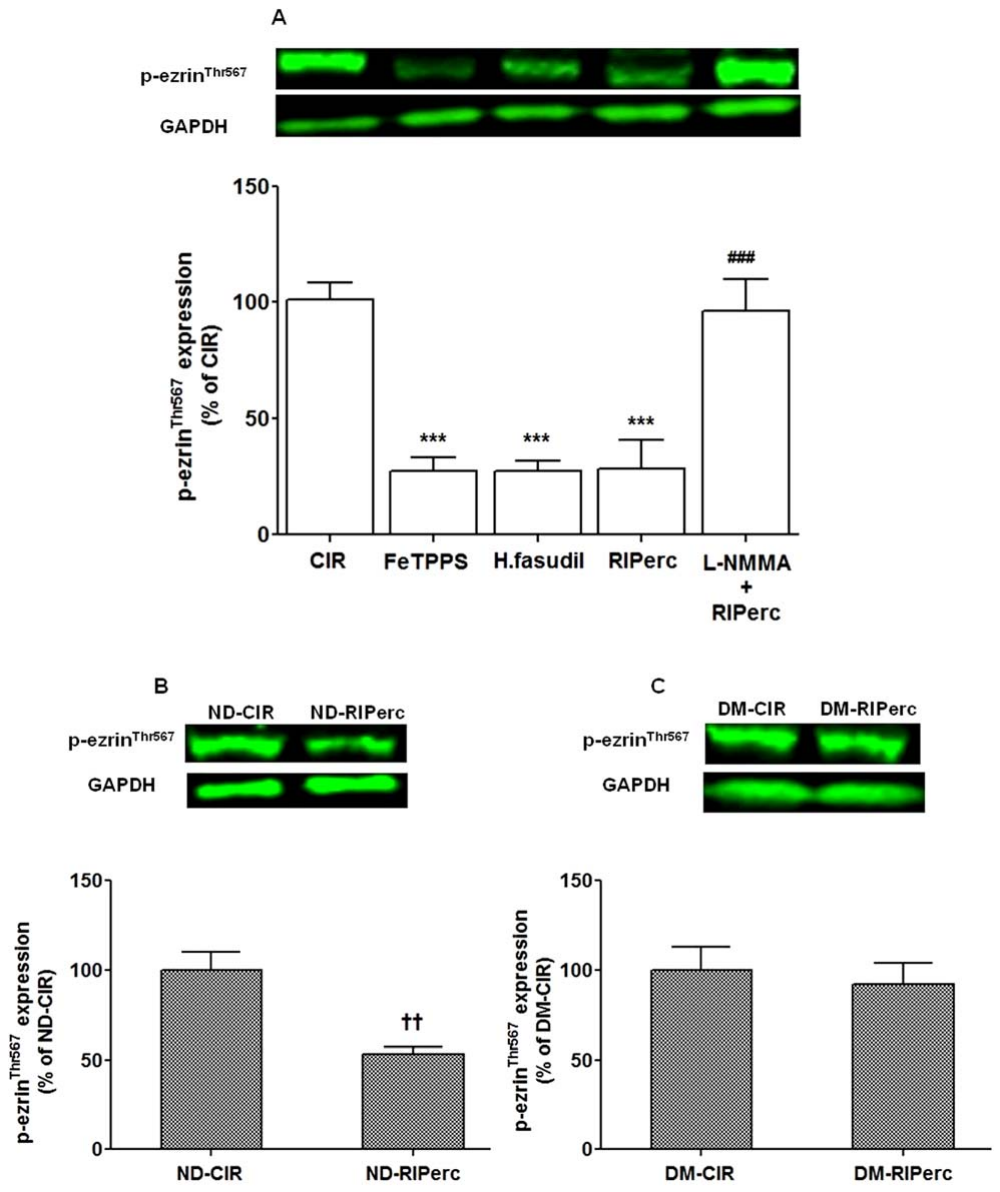
ROCK activity expressed as a phosphorylation of ezrin following ischemia/reperfusion. (A) Effect of the peroxynitrite decomposition catalyst FeTPPS, the ROCK inhibitor hydroxyfasudil (H.fasudil), remote ischemic preconditioning (RIPerc) and RIPerc+the NOS inhibitor L-NMMA in protocol 1. (B and C) Effect of RIPerC in non-diabetic and diabetic rats of protocol 2. Values are means ± SEM; n = 5–7. ***P<0.001 vs. CIR; ^###^ *P*<0.001 vs. RIPerc and ^††^ *P*<0.01 vs. ND-CIR.

The effect of peroxynitrite and ROCK activity were determined by the peroxynitrite decomposition catalyst FeTPPS and the ROCK inhibitor hydroxyfasudil, respectively. There was no difference in the area at risk between groups (Fig. 1A). Infarct size was significantly reduced by both FeTPPS and hydroxyfasudil (51±4% and 49±3% of the area at risk, respectively) in comparison with the CIR group (69±2%, Fig. 1C). FeTPPS and hydroxyfasudil markedly suppressed arginase activity in tissue samples taken from the area at risk of the left ventricle following IR (Fig. 2A, *P*<0.001 vs. CIR) without affecting expressions of either arginase 1 or 2 protein (Fig. 2B and 2C). By contrast, arginase activity in non-ischemic myocardium was unaffected by either FeTTPS or hydroxyfasudil (data not shown). In addition, FeTPPS suppressed the phosphorylated form of ezrin as a marker of ROCK activity in compared to controls following IR by a magnitude similar to that induced by the ROCK inhibitor (Fig. 3A, *P*<0.001).

#### 3.2.2 Effect of RIPerc on infarct size and ROCK–arginase activity

RIPerc reduced infarct size by a magnitude comparable to that induced by FeTTPS and hydroxyfasudil (Fig. 1C). In addition, RIPerc markedly reduced arginase activity (Fig. 2A) and ROCK activity (Fig. 3A). These effects of RIPerc were completely abolished by administration of L-NMMA (Figs. 1C, 2A and 3A).

#### 3.2.3 Effect of RIPerc on eNOS phoshorylation and peroxynitrite formation

**Figure 4:**
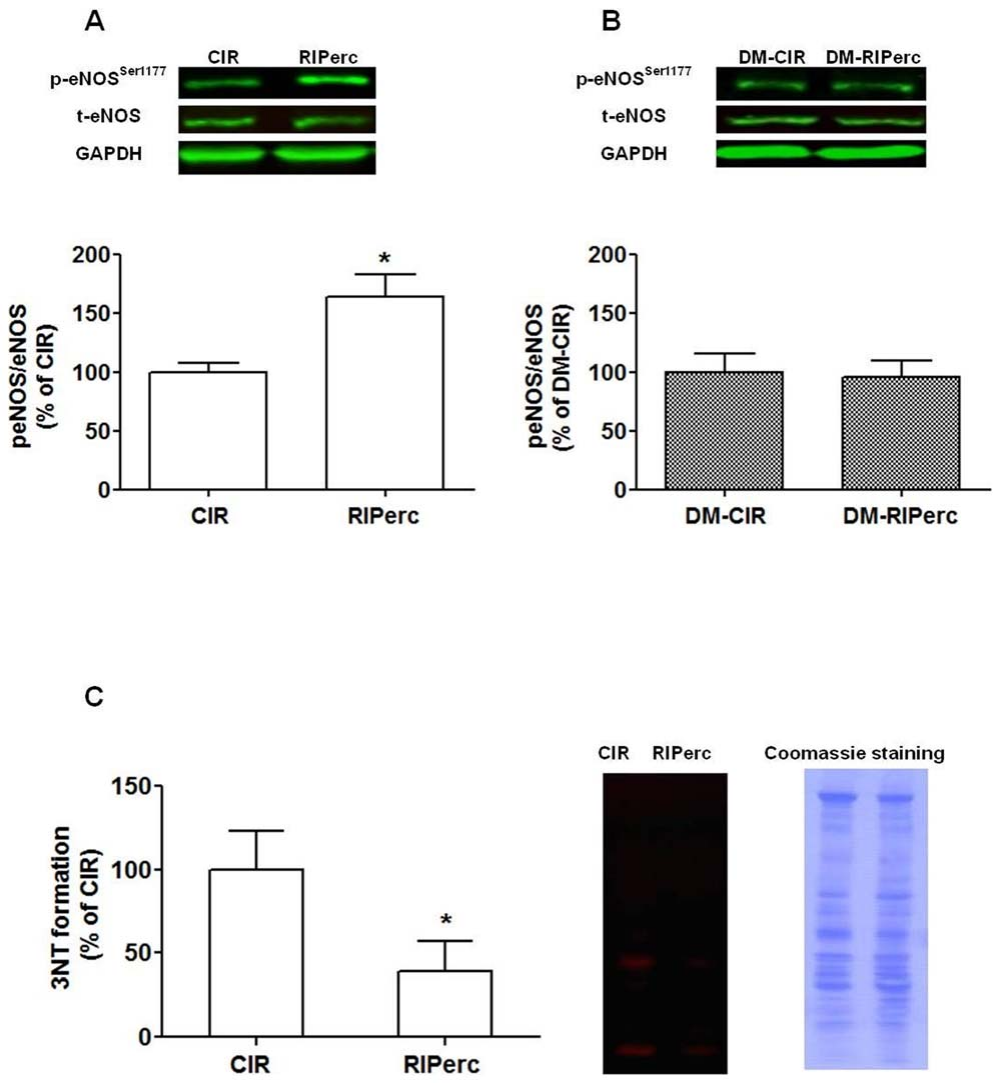
The effect of RIPerc on eNOS expression and peroxynitrite formation. (A and C) Effect of remote ischemic perconditioning (RIPerc) on phosphorylated of eNOS at Ser1177 (p-eNOS), total eNOS and nitrotyrosine (3NT) in protocol 1. (B) Effect of RIPerc on NOS expression in non-diabetic and diabetic rats of protocol 2. Values are means ± SEM; n = 5–8. *P<0.05 vs. CIR.

Total and phosphorylated (Ser1177) eNOS was determined in left ventricle tissue samples taken from the area at risk from CIR and RIPerc groups. RIPerc enhanced phoshorylation of eNOS at Ser1177 following IR (Fig. 4A). In addition, the level of 3NT, a marker of peroxynitrite formation, was markedly attenuated by RIPerc (Fig. 4C).

### 3.3 Results of protocol 2

#### 3.3.1 Characteristics of type 1 diabetes

Blood glucose level was 27.3±1.0 mmol/l four weeks after the administration of streptozotocin compared to 4.9±0.2 mmol/l in the control group (*P*<0.001). Body weight was significantly lower in the diabetic group (291±12 g) than in the control group (394±9 g, *P*<0.001).

#### 3.3.2 Infarct size in protocol 2

Infarct size was similar in non-diabetic and diabetic animals subjected to IR (Fig. 1D). As in animals included in protocol 1, RIPerc resulted in a significant reduction of infarct size in the non-diabetic group (Fig. 1D). In contrast, RIPerc failed to reduce infarct size in rats with type 1 diabetes in comparison with controls (68±2% of area at risk in DM-CIR: vs. 63±2% in DM-RIPerc; Fig. 1D).

#### 3.3.3 Arginase expression and ROCK–arginase activity following IR in diabetes

**Figure 5:**
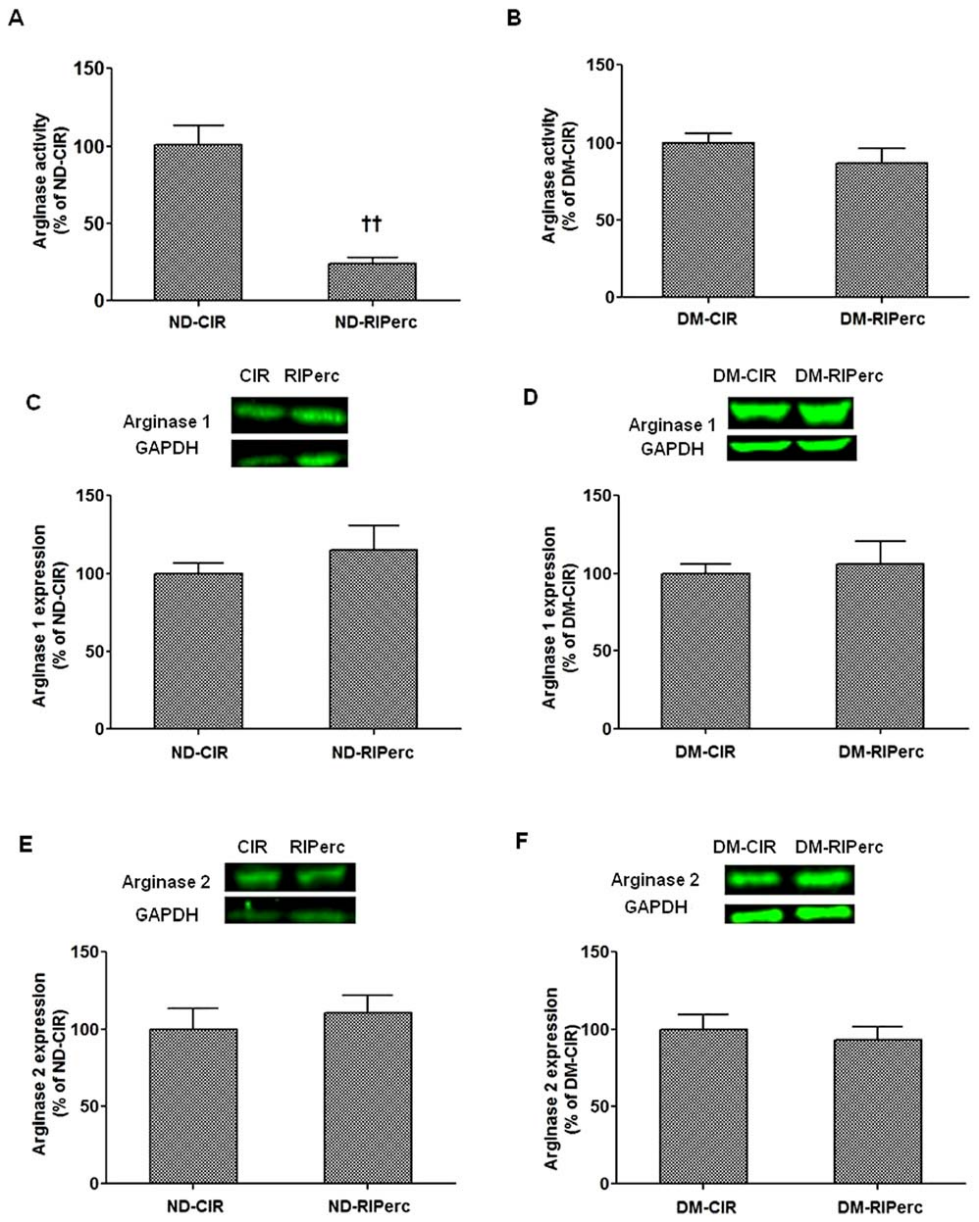
Arginase activity and protein expression in protocol 2. (A and B) Arginase activity and (C–F) expression of arginase 1 and 2 in non-diabetic rats (ND) and rats with type 1 diabetes mellitus (DM). Values are means ± SEM; n = 5–7 ^††^ *P*<0.01 vs. ND-CIR.

Arginase activity in non-ischemic myocardium was increased by 48±11% (*P*<0.05) in rats with type 1 diabetes compared to non-diabetic controls. Furthermore, arginase activity was increased in myocardium from the area at risk (ischemic) of rats with diabetes (by 70±20%) and in non-diabetic control rats (by 87±11%), (*P*<0.01) in comparison with non-ischemic myocardium. In tissue samples from the area at risk of the left ventricle arginase activity was significantly reduced by RIPerc in the non-diabetic control rats (Fig. 5A). By contrast, RIPerc did not affect arginase activity rats with diabetes (Fig. 5B). The expressions of arginase 1 or 2 protein were unaffected by RIPerc (Fig. 5C–F). Moreover, in contrast to the marked changes observed in non-diabetic rats, RIPerc failed to reduce ROCK activity or to increase the expression of phosphorylated eNOS at Ser1177 in rats with diabetes (Figs. 3 and 4).

## Discussion

The main findings of the current study are that (1) upregulation of arginase activity by IR is mediated by peroxynitrite and ROCK activity in the myocardium, (2) RIPerc induced by bilateral femoral artery occlusion during sustained myocardial ischemia protects the heart against IR injury via a mechanism involving attenuated peroxynitrite formation and ROCK signaling, (3) the inhibition of this pathway results in downstream attenuation of arginase and increased NOS activity, and (4) the cardioprotective effect as well as the associated down-regulation of ROCK and arginase activity induced by RIPerc is abolished in a model of type 1 diabetes.

There is strong evidence that myocardial IR-injury is critically dependent on enhanced arginase activity resulting in reduced NO production and bioavailability [3], [4], [5]. In addition, available evidence suggests that arginase activity is significantly increased in the myocardium at risk already within 20 min after the onset of reperfusion [25]. Although the protective effect of arginase inhibition under IR has been confirmed, the exact mechanism regarding the upregulation of arginase activity in the myocardium is still unknown. Data obtained in cultured endothelial cells suggest that peroxynitrite is an upstream mediator of ROCK and arginase activation [8], [9]. Since both peroxynitrite and ROCK activity are increased upon reperfusion and contribute to myocardial IR injury [26], [27], we hypothesized that peroxynitrite and ROCK are possible upstream activators of arginase in the myocardium during IR *in vivo*. We demonstrate that administration of the peroxynitrite decomposition catalyst FeTPPS prior to the onset of reperfusion significantly reduced both ROCK and arginase activity, indicating that peroxynitrite is an activator of ROCK and arginase during myocardial IR. Furthermore, inhibition of ROCK suppressed arginase activity during myocardial IR. These findings are agreement with previous data from cell culture studies [8] and suggest that peroxynitrite-induced ROCK signaling stimulates arginase activity in the myocardium during IR. Although FeTPPS and hydroxyfasudil attenuated arginase activity, expression of arginase 1 and 2 protein was unaffected. In accordance with previous results [4] this indicates that alterations in myocardial arginase activity are not critically dependent on changes in arginase protein levels.

Next we investigated whether the peroxynitrite-ROCK-arginase-NO pathway is involved in the cardioprotective effect mediated by RIPerc. The reduction in infarct size induced by RIPerc was associated with a down-regulation of ROCK activity, which is in line with a previous observation [14]. In addition, we demonstrate that RIPerc reduced the 3NT-levels reflecting attenuated peroxynitrite formation. Based on the finding that the peroxynitrite-ROCK pathway activate arginase in IR, we hypothesized that arginase may be down-regulated by RIPerc. Accordingly, the endogenous cadioprotective mechanism triggered by RIPerc reduces myocardial arginase activity following IR. Since both inhibition of ROCK and arginase is known to result in increased eNOS activity [3], [28], [29] and enhanced eNOS substrate availability, we explored the effect of RIPerc following NOS inhibition and its effect on eNOS phosphorylation. In accordance with previous studies demonstrating the protective effect of eNOS and NO against myocardial IR injury [30], [31], we found that RIPerc increased eNOS phosphorylation at Ser1177. Furthermore, the cardioprotective effect of RIPerc was completely abolished by the NOS inhibitor L-NMMA, confirming involvement of NOS. Since previous studies have shown that arginase inhibition protects from myocardial IR injury by a mechanism dependent on NOS activity and bioavailability of NO [3], [4], [5], it is tempting to speculate that down regulation of arginase is involved in the cardioprotective effect mediated by RIPerc. This needs to be established in future studies, however.

In this study, we demonstrate that RIPerc failed to induce cardioprotection in a rat model of type 1 diabetes. It is well established that the sensitivity of the diabetic heart to local pre- and postconditioning is impaired [32], but this is to the best of our knowledge the first demonstration that the cardioprotective effect of remote ischemic perconditioning is lost in diabetes. This may, at least in part, be associated with the dysregulation of signaling pathway activating eNOS [16], [32]. In accordance, we found that myocardial eNOS (Ser1177) phosphorylation was increased in non-diabetic rats but unaffected by RIPerc in diabetic rats. Previous studies showed that increased arginase and ROCK activity are involved in diabetes-induced vascular dysfunction via mechanisms, at least in part, associated with reduction in NO bioavailability [2], [33]. It has further been demonstrated that the Rho/ROCK signaling pathway is enhanced in the diabetic myocardium [34]. In the present study we show that myocardial arginase activity is increased rats with type 1 diabetes compared to non-diabetic controls. Interestingly, both arginase and ROCK activity was unchanged by RIPerc in the diabetic group, which is in sharp contrast to the down-regulation of arginase and ROCK activity by of RIPerc in non-diabetic rats. It remains to be established whether diabetes-induced upregulation of ROCK and arginase activity contribute to failure of RIPerc to induce cardioprotection in type 1 diabetes.

There are some limitations with the present study that deserve consideration. First, L-NMMA completely abolished the cardioprotective effect of RIPerc. We did not include a group receiving L-NMMA alone since it previously has been shown that L-NMMA and other NOS inhibitors do not influence IR injury *per se* [21], [22], [23]. Second, the current study demonstrates that reduced arginase and ROCK activity is associated with the cardioprotective effect of RIPerc, but the data do not provide evidence regarding a causative effect. Additional studies are needed to confirm whether this mechanism is a mediator of cardioprotection by RIPerc. Third, although our data demonstrate loss of cardioprotection induced by RIPerc in a model of type 1 diabetes, further studies are needed to establish whether similar effect are obtained in other models of diabetes including type 2 diabetes. Fourth, further studies are needed to clarify if the loss of the cardioprotective effect of RIPerc in type 1 in diabetes is due to impairment of signal transduction from the remote organ to the heart and/or impairment of protective signaling pathways in heart and their relation to arginase and ROCK activity.

In conclusion, the present study demonstrates that peroxynitrite and ROCK signaling pathways are involved in the upregulation of arginase activity during myocardial IR. In addition, we propose that reduction in arginase activity mediated by reduced formation of peroxynitrite and ROCK activity is of importance for the NOS-dependent cardioprotective effect of RIPerc. Importantly, our study also demonstrates that the cardioprotective effect and the associated signaling effects on arginase, ROCK and NOS by RIPerc are abolished in a model of type 1 diabetes.
